# Supplementary figures and images for: Mental and substance use disorders and food insecurity among homeless adults participating in the At Home/Chez Soi study
Source: PLoS One. 2020 Apr 23;15(4):e0232001. doi: 10.1371/journal.pone.0232001 (PMC7179857; doi:10.1371/journal.pone.0232001)

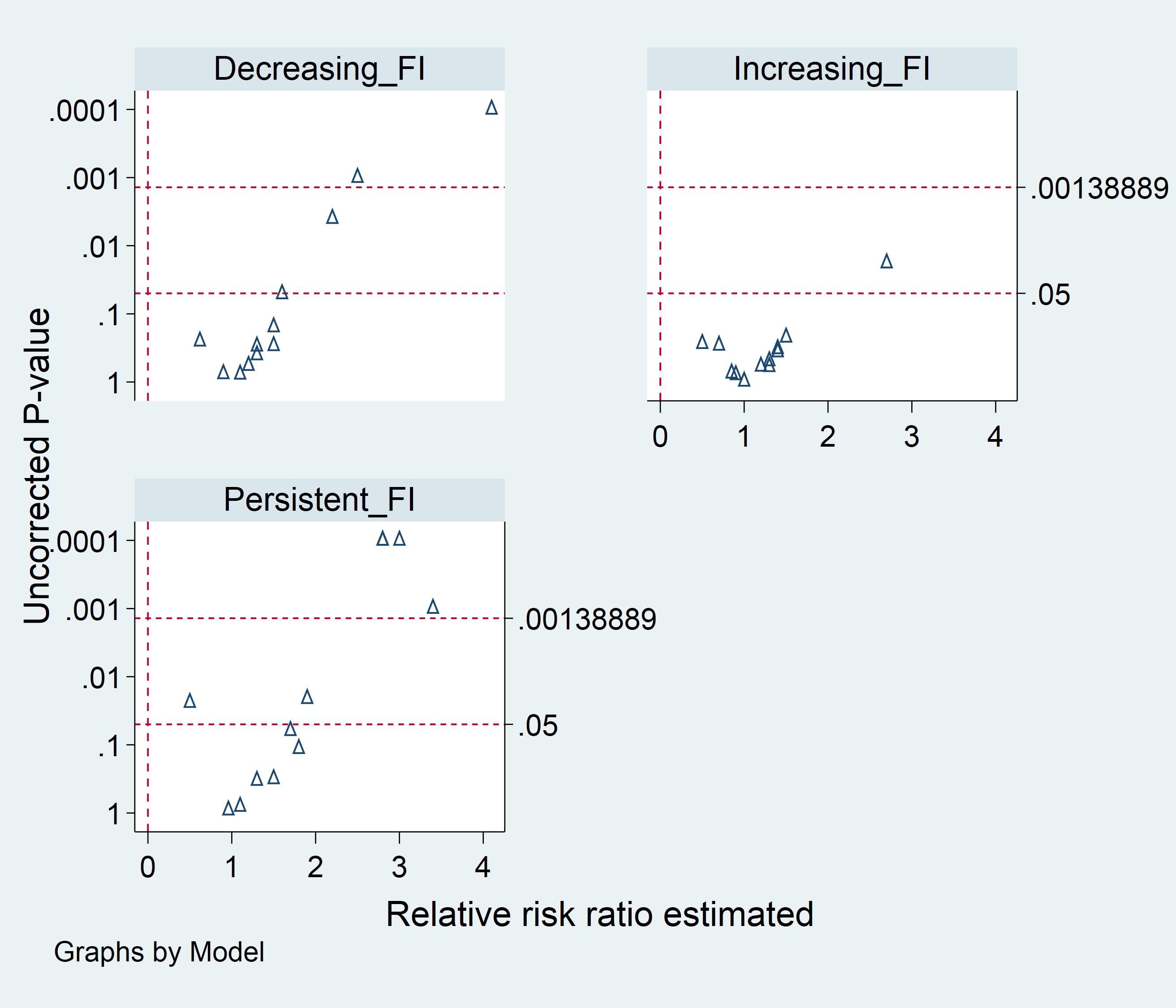


Fig A1: Smile plot of Relative risk ratio by food security trajectory groups

Supplement: S1 Fig — (DOCX) [file pone.0232001.s001.docx]
