# Supplementary material for: Mental and substance use disorders and food insecurity among homeless adults participating in the At Home/Chez Soi study
Source: PLoS One. 2020 Apr 23;15(4):e0232001. doi: 10.1371/journal.pone.0232001 (PMC7179857; doi:10.1371/journal.pone.0232001)
Supplement: S1 Table — (DOCX) [file pone.0232001.s002.docx]

**Table A1: Fisher Test comparing characteristics between Study Participants (n=520) and the Excluded group (n=55)**

| **Characteristics** | **Excluded group**  **(n=55)** | **Study Particpants (n=520)** | **P-value** |
| --- | --- | --- | --- |
| **Age(mean[SD]) ˨** | 41.1[1.885] | 40.2[0.509] | 0.634 |
| **Gender** |  |  |  |
| Male | 70.08 | 72.34 |  |
| Female | 29.92 | 27.66 | 0.745 |
| **Self-identified ethnic group** | |  |  |
| White | 65.15 | 78.72 |  |
| non-white/ethnic groups | 34.85 | 21.28 | 0.059 |
| **Level of need** |  |  |  |
| Moderate need | 67.05 | 51.06 |  |
| High need | 32.95 | 48.94 | **0.027** |
| **Intervention group** | |  |  |
| TAU | 47.16 | 53.19 |  |
| AF | 52.84 | 46.81 | 0.428 |

˨ Student’s test

Bold P-value means statistically significant at a level of confidence of 95%
