# Supplementary material for: Mental and substance use disorders and food insecurity among homeless adults participating in the At Home/Chez Soi study
Source: PLoS One. 2020 Apr 23;15(4):e0232001. doi: 10.1371/journal.pone.0232001 (PMC7179857; doi:10.1371/journal.pone.0232001)
Supplement: S2 Table — (DOCX) [file pone.0232001.s003.docx]

**Table A2: Means (and standard deviation) of the number of food insecurity assessments of the participants included in this analysis by intervention group and by key mental health disorders**

| **Characteristics** | **Mean (SD)** | **P-value** |
| --- | --- | --- |
| **Intervention group** |  |  |
| TAU | 3.1 (0.110) |  |
| HF | 3.6 (0.084) | **0.0018** |
| Major depressive episode |  |  |
| No | 3.3 (0.088) |  |
| Yes | 3.5 (0.108) | 0.0674 |
| Manic or Hypomanic episode |  |  |
| No | 3.4 (0.071) |  |
| Yes | 3.4 (0.232) | 0.9059 |
| PTSD |  |  |
| No | 3.3 (0.078) |  |
| Yes | 3.5 (0.140) | 0.3485 |
| Panic disorder |  |  |
| No | 3.3 (0.073) |  |
| Yes | 3.6 (0.186) | 0.1228 |
| Mood disorder with psychotic features | |  |
| No | 3.3 (0.077) |  |
| Yes | 3.5 (0.150) | 0.4792 |
| Psychotic disorder |  |  |
| No | 3.5 (0.085) |  |
| Yes | 3.2 (0.115) | 0.0697 |
| Alcohol disorder |  |  |
| No | 3.2 (0.083) |  |
| Yes | 3.7 (0.114) | 0.0009 |
| Substance disorder |  |  |
| No | 3.3 (0.087) |  |
| Yes | 3.5 (0.111) | 0.0487 |
| Suicidality |  |  |
| No | 3.1 (0.115) |  |
| Yes | 3.5 (0.084) | 0.0045 |
