# Supplementary material for: Mental and substance use disorders and food insecurity among homeless adults participating in the At Home/Chez Soi study
Source: PLoS One. 2020 Apr 23;15(4):e0232001. doi: 10.1371/journal.pone.0232001 (PMC7179857; doi:10.1371/journal.pone.0232001)
Supplement: S3 Table — (DOCX) [file pone.0232001.s004.docx]

**Table A3. Participants’ Characteristics at baseline by Intervention group**

| **Variable** | **Total (n=520)** | **HF (n=282)** | **TAU (n=238)** | **P-value** |
| --- | --- | --- | --- | --- |
|  |  |  |  |  |
| Female | 165 (31.7) | 95 (33.7) | 70 (29.4) | 0.297 |
| Age (mean(SD)) ˨ | 40.3 (11.8) | 39.4 (11.4) | 41.3 (12.1) | **0.0508** |
| ***education level*** |  |  |  |  |
| Middle/high shool | 241 (46.3) | 138 (48.9) | 103 (43.3) |  |
| Completed high school | 94 (18.1) | 46 (16.3) | 48 (20.2) |  |
| graduate/posgraduate | 170 (32.7) | 94 (33.3) | 76 (31.9) | 0.162^¥^ |
| Missing | 15 (2.9) | 5 (1.8) | 10 (4.2) |  |
| **Self-identified ethnic group (white vs non-white/ethnic groups)** | 304 (58.5) | 170 (60.3) | 134 (56.3) | 0.359 |
| ***Level of need*** |  |  |  |  |
| High level | 175 (33.7) | 89 (31.6) | 86 (36.1) |  |
| Low level | 345 (66.3) | 193 (68.4) | 152 (63.9) | 0.198 |
| ***Lifetime homelessness*** |  |  |  |  |
| Less than 3 years | 225 (43.3) | 130 (46.1) | 95 (39.9) |  |
| More than 3 years | 270 (51.9) | 141 (50.0) | 129 (54.2) |  |
| Missing | 25 (4.8) | 11 (3.9) | 14 (5.9) | 0.268 |
| ***Mental illness*** |  |  |  |  |
| Major depressive episode | 187 (36) | 103 (36.5) | 84 (35.3) | 0.771 |
| Manic or Hypomanic episode | 57 (11) | 37 (13.1) | 20 (8.4) | 0.086 |
| PTSD | 126 (24.2) | 73 (25.9) | 53 (22.3) | 0.337 |
| Panic disorder | 74 (14.2) | 41 (14.5) | 33 (13.9) | 0.827 |
| Mood disorder with psychotic features | 107 (20.6) | 58 (20.6) | 49 (20.6) | 0.995 |
| Psychotic disoder | 189 (36.3) | 103 (36.5) | 86 (36.1) | 0.928 |
| Alcohol dependence | 151 (29) | 74 (26.2) | 77 (32.4) | 0.126 |
| Substance disorder | 197 (37.9) | 107 (37.9) | 90 (37.8) | 0.976 |
| Alcohol abuse | 73 (14.0) | 45 (16.0) | 28 (11.8) | 0.170 |
| Substance abuse | 50 (9.6) | 31 (11.0) | 19 (8.0) | 0.246 |
| Suicidality | 345 (66.3) | 180 (63.8) | 165 (69.3) | 0.186 |
| COD | 246 (47.3) | 128 (45.4) | 118 (49.6) | 0.340 |

˨ Student’s test

Bold P-value means close to be statistically significant at a level of confidence of 95%

^¥^ Test was computed after excluding the missing value
