# Supplementary material for: Mental and substance use disorders and food insecurity among homeless adults participating in the At Home/Chez Soi study
Source: PLoS One. 2020 Apr 23;15(4):e0232001. doi: 10.1371/journal.pone.0232001 (PMC7179857; doi:10.1371/journal.pone.0232001)
Supplement: S4 Table — (DOCX) [file pone.0232001.s005.docx]

**Table A4. Participants’ Characteristics at baseline by Food Security Trajectory Group**

| **Variable** | **Total (n=520)** | **Stable High (n=140)** | **Medium to high (n=75)** | **Medium to low (n=169)** | **Stable low (n=136)** |
| --- | --- | --- | --- | --- | --- |
|  |  |  |  |  |  |
| Female | 165 (31.7) | 43 (30.7) | 23 (30.7) | 51 (30.2) | 48 (35.3) |
| Age: mean(standard deviation) | 40.3 (11.8) | 39.7 (10.4) | 40.7 (12.2) | 40.1 (11.8) | 40.8 (12.8) |
| ***education level*** |  |  |  |  |  |
| Middle/high school | 241 (46.3) | 59 (42.1) | 40 (53.3) | 80 (47.3) | 62 (45.6) |
| Completed high school | 94 (18.1) | 26 (18.6) | 18 (24) | 31 (18.3) | 19 (14) |
| graduate/postgraduate | 170 (32.7) | 49 (35) | 17 (22.7) | 55 (32.5) | 49 (36) |
| Missing | 15 (2.9) | 6 (4.3) | (0) | 3 (1.8) | 6 (4.4) |
| **Self-identified ethnic group (white vs non-white/ethnic groups)** | 304 (58.5) | 89 (63.6) | 38 (50.7) | 91 (53.8) | 86 (63.2) |
| ***Level of need*** |  |  |  |  |  |
| High level | 175 (33.7) | 46 (32.9) | 28 (37.3) | 57 (33.7) | 44 (32.4) |
| Low level | 345 (66.3) | 94 (67.1) | 47 (62.7) | 112 (66.3) | 92 (67.6) |
| ***Lifetime homelessness*** |  |  |  |  |  |
| Less than 3 years | 225 (43.3) | 55 (39.3) | 30 (40) | 75 (44.4) | 65 (47.8) |
| More than 3 years | 270 (51.9) | 76 (54.3) | 45 (60) | 87 (51.5) | 62 (45.6) |
| Missing | 25 (4.8) | 9 (6.4) | 0 (0) | 7 (4.1) | 9 (6.6) |
| ***Intervention*** |  |  |  |  |  |
| At Home | 282 (54.2) | 73 (52.1) | 40 (53.3) | 93 (55) | 76 (55.9) |
| Treatment as Usual | 238 (45.8) | 67 (47.9) | 35 (46.7) | 76 (45) | 60 (44.1) |
| ***Mental illness*** |  |  |  |  |  |
| Major depressive episode | 187 (36) | 59 (42.1) | 27 (36) | 61 (36.1) | 40 (29.4) |
| Manic or Hypomanic episode | 57 (11) | 16 (11.4) | 5 (6.7) | 21 (12.4) | 15 (11) |
| Posttraumatic disorder | 126 (24.2) | 40 (28.6) | 14 (18.7) | 46 (27.2) | 26 (19.1) |
| Panic disorder | 74 (14.2) | 21 (15) | 11 (14.7) | 27 (16) | 15 (11) |
| Mood disorder with psychotic features | 107 (20.6) | 33 (23.6) | 16 (21.3) | 46 (27.2) | 12 (8.8) |
| Psychotic disorder | 189 (36.3) | 42 (30) | 25 (33.3) | 65 (38.5) | 57 (41.9) |
| Alcohol disorder | 151 (29) | 42 (30) | 24 (32) | 53 (31.4) | 32 (23.5) |
| Substance disorder | 197 (37.9) | 66 (47.1) | 26 (34.7) | 72 (42.6) | 33 (24.3) |
| Suicidality | 345 (66.3) | 106 (75.7) | 49 (65.3) | 115 (68) | 75 (55.1) |
